# The African Light Source: history, context and future

**DOI:** 10.1107/S1600577523009682

**Published:** 2024-01-01

**Authors:** Simon H. Connell, Kathleen Dollman, Gihan Kamel, Sameen A. Khan, Edward Mitchell, Sekazi K. Mtingwa, Marcus C. Newton, Prosper Ngabonziza, Emmanuel Nji, Lawrence Norris, Michele Zema

**Affiliations:** a Department of Mechanical Engineering Science, University of Johannesburg, South Africa; b ESRF – The European Synchrotron, Grenoble, France; c SESAME – Synchrotron light for Experimental Science and Applications in the Middle East, Allan, Jordan; d Department of Physics, Faculty of Science, Helwan University, Cairo, Egypt; e Dhofar University, Salalah, Sultanate of Oman; f TriSEED Consultants, LLC, North Carolina, USA; g Physics and Astronomy, University of Southampton, Southampton, United Kingdom; h Department of Physics and Astronomy, Louisiana State University, USA; i Department of Physics, University of Johannesburg, South Africa; j Centre for Research in Therapeutic Sciences (CREATES), Strathmore University, Madaraka Estate, Ole Sangale Road, 59857-00200 Nairobi, Kenya; k BioStruct-Africa, Vårby, Stockholm, Sweden; l African Physical Society, Senegal; m International Union of Crystallography, Chester, United Kingdom; n University of Bari ‘Aldo Moro’, Bari, Italy; ESRF – The European Synchrotron, France

**Keywords:** African Light Source, AfLS, synchrotron, history

## Abstract

The African Light Source (AfLS) project is now almost eight years old. This article assesses the history, current context and future of the project. There is by now considerable momentum in building the user community, including deep training, facilitating access to current facilities, growing the scientific output, scientific networks and growing the local laboratory-scale research infrastructure. The Conceptual Design Report for the AfLS is in its final editing stages. This document specifies the socio-economic and scientific rationales and the technical aspects amongst others. The AfLS is supported by many national and Pan-African scientific professional bodies and voluntary associates across many scientific disciplines, and there are stakeholders throughout the continent and beyond. The current roadmap phases have expanded to include national and Pan-African level conversations with policy makers through new Strategic Task Force groups. The document summarizes this progress and discusses the future of the project.

## Abstract 2

The African Light Source project towards a light source for the African continent is described.

## 1. The African Light Source: context, large scale infrastructures, science diplomacy

Science is widely regarded as the engine of new knowledge generation, novel technologies, innovation, wealth generation, socio-economic development, even the engine of democracy. It is also the best hope for achieving sustainable human life on earth and beyond. The development of human civilization is beset with challenges. The set of challenges to be addressed is often articulated in the 17 Sustainable Development Goals (SDGs) adopted by the United Nations (The UN-SDGs, 2015 ▸). It is only through constant research and innovation that we can begin to understand and address these challenges.

As of today, more than 55 synchrotron radiation facilities are operating in 26 countries. All habitable continents are represented except for Africa (Khan, 2022 ▸). Most of the developed world has such facilities and some developing countries also have synchrotrons. About half of the synchrotrons, or, more generally, advanced light sources (AdLSs), have or plan upgrades from third-generation to fourth-generation facilities. The fourth-generation facilities have orders of magnitude better performance for research and discovery. They address an increasingly wide range of disciplines and industrial applications. An AdLS is clearly much prioritized as a necessary large-scale research infrastructure by a majority of nations.

International science institutions played a significant role in the rebuilding of Europe after the second world war. Notable among these is the European Laboratory for Particle Physics (CERN) in Geneva, Switzerland. Founded in 1954, CERN was one of Europe’s first joint ventures and now includes 22 member states. The budget for establishing and running CERN is borne by the member countries according to a standard national formula. CERN’s accomplishments include: the ubiquitous world wide web; grid computing, which has applications in several areas including climate studies and genome analysis; and electronic detectors with applications in medical X-ray imaging. Scientific collaboration beyond national boundaries has been a force for peace since before the cold war. The international science centre located in Jordan, and known by the acronym SESAME (Synchrotron-light for Experimental Science and Applications in the Middle East), is promoting science and fostering international cooperation in the Middle East. Very much like CERN, SESAME is under the very valuable political umbrella of UNESCO. The success of CERN in terms of science diplomacy and the value of an international facility played a role in the creation of the European Synchrotron Radiation Facility (ESRF) in 1974 in Grenoble, France. The ESRF is a jointly operated facility with the support of 21 countries from Europe and beyond, including South Africa.

Science is a cooperative adventure and research infrastructures such as AdLSs have become ubiquitous in their role as coordinated transversal hubs of science and technology, traversing cultural and national barriers. The world, its regions and nations depend on access to these high-quality large-scale research infrastructures in order to carry out high-impact and necessary research across a diverse set of scientific disciplines and at the highest level. Indeed, an advanced synchrotron light source stands out as a large-scale research infrastructure which can provide access to unique scientific data for much of this research.

In Europe alone, light sources serve a vibrant community of about 30000 researchers from academia and industry, with the portfolio of synchrotrons undergoing substantial updates starting with the European Synchrotron Radiation Facility that was recently upgraded to become the first high-energy fourth-generation synchrotron and, as mentioned above, with now many other upgrades being planned at other light sources in Europe and worldwide.

Seeking to change the lagging position of Africa in this respect and to drive an AdLS design, construction and operation for Africa, in Africa, and by Africa, are overarching goals of the African Light Source Foundation (AfLS) (The African Light Source, 2015 ▸). The AfLS is at the heart of a large community-driven movement for an AdLS in Africa and which led to the creation of the AfLS as a community mandated body to develop and progress a roadmap towards an AdLS for Africa.

Most recently, the AfLS has prepared an in-depth Conceptual Design Report, soon to be released, which positions an AdLS as one of the premier large-scale research facilities that supports much of the science required for addressing the challenges in the 17 SDGs. A fourth-generation synchrotron has emerged as the best candidate for Africa, with a full suite of beamlines and capacity for science in many disciplines, aimed as a facility of global stature.

## 2. Motivation for the AfLS

An AdLS is a premier multidisciplinary large-scale research infrastructure, probably best described as a super-microscope. As mentioned above, it is a polyvalent facility, benefiting numerous scientific disciplines, manufacturing industries and hi-tech businesses.

With an AdLS at the heart of many scientific ecosystems, ownership of an AdLS is driven by the return that the research infrastructure can provide in enabling Africa to take the lead in solving challenges of particular relevance to Africa, or where Africa has a natural niche for excellence, where it has natural resources – raw materials, biodiversity or supporting agriculture, water supply, energy provision, as well as health and fundamental science to drive future careers and opportunities. To date, Africa lacks an AdLS; thus, Africans must travel and perform ‘suitcase science’, with many becoming science diasporeans. African scientists are involved in excellent research in a range of disciplines at AdLSs outside Africa, where they develop global research networks and collaborations.

More and more, emerging young African scientists trained in this context gain prestigious positions abroad, thereby joining the African science diaspora. This is not necessarily bad; it is positive that African scientists perform at global levels. This global participation and cooperation in science must continue even after Africa has its own AdLS. The point is that Africa prefers and deserves a fully bi-directional global participation. Africa prefers to play a greater role in determining the research agenda, especially where it affects Africa itself, considering that African-based scientists generate <1% of research publications. Africa has its own research drivers, with examples in health, palaeontology, novel materials, mineral beneficiation, sustainable environment, big data analytics and leapfrog technologies. The Global North research issues are not always aligned with the research priorities of Africa.

There is also the issue of increasing Africa’s intellectual share of technological developments, innovation and competitive industry. To pursue these, Africa needs a direct involvement in an AdLS facility, not just as an external user. The AfLS is receiving policymaker and stakeholder feedback to raise the priority of engineer and technologist training and to develop local capacity in both human and infrastructural resources. This growth in expertise and capability will go on to feed the capacity for industry to be competitive and for African home-grown and home-owned innovations.

In the coming few decades, Africa will become home to the bulk of the world’s youth. Already most of the African population are under the age of 30. This makes an urgent call for Africa to invest more in science, technology and innovation, particularly in building an AdLS as a scientific, technological and higher-education hub for the continent. This energetic and young African population may then not only be productive for the continent but also be an opportunity to be seized for Africa in addressing global challenges through scientific and technological innovations.

The existence of an AfLS is good for the whole continent, not just the single country that succeeds in the bid process. It means new capacity building and new associated dispersed research infrastructures, and increased positions at African universities will be seen Africa-wide, not only those nearest to the new large-scale infrastructure. These broad socio-economic impacts at regional, national and international levels are an important component of the return on investment in such large-scale facilities. The UK’s DIAMOND synchrotron light source has been calculated to have delivered at least £1.8 billion cumulative monetized impact in 2021 (UKRI, 2021 ▸) which compares very favourably with the £2.6 billion investment made in the facility to date (UKRI, 2022 ▸), such that the British synchrotron has cost taxpayers less than one cup of coffee each per year. In addition, technology now makes large-scale infrastructure accessible remotely, lowering travel barriers and related carbon emissions. All of Africa will benefit from the AfLS, not just the hosting country. It will be a win for all Africa.

Finally, there is the culture of learning and innovation, and the role of science and technology as perceived by society, especially by the upcoming generation who will become the scientific and political leaders of the future. Here, high-profile, large-scale projects play a key role. Africa needs to have its own high-performance AdLS research capacity for this sociological and inspirational reason as well.

A survey of the penetration of the use of an AdLS as a tool in science, or even the use of X-rays as a tool, shows the size of the possible user base of a future AfLS. This survey is very difficult, as there is no single convenient source of information, and much of Africa’s research is done in the science diaspora. The heritage sciences, especially palaeontology, have been a niche research area for Africa. A recent review (von der Heyden *et al.*, 2020 ▸) has chronicled a footprint in Africa of 19 countries with some 80 publications. This is likely to be a conservative estimate. Some highlights from this field would be the phase-contrast tomographic study of the *Australopithecus sediba* MH1 cranium which revealed the shape of the endocranium cavity (Carlson *et al.*, 2011 ▸), and a similar study of an early triassic fossilized burrow cast which very unusually contained an injured temnospondyl amphibian sheltering with an aestivating therapsid (Fernandez *et al.*, 2013 ▸). The same review also surveys synchrotron studies on African geological and environmental geochemistry sample material and finds a footprint in 12 African countries with more than twice this amount of papers (von der Heyden *et al.*, 2020 ▸). These are dominated by the various spectroscopies, but there are also diffraction techniques and imaging. The footprint of the bio-science capacity is shown by Connell *et al.* (2019 ▸), where more than 20 centres are identified. A significant training exercise known as START partners Africa with DIAMOND (Nicklin *et al.*, 2022 ▸). In the bioscience research area it covered the topics of SARS-CoV-2, snakebite envenomation, HIV, tuberculosis, malaria, bilharzia, human papilloma virus, cardiovascular disease, human metabolic disorders, African horse sickness virus, as well as industrial enzymes that can be used for the manufacture of medicines and commodity chemicals. Many of these programmes predated START and continue. Several materials science research programmes are also mentioned here. The review (Connell *et al.*, 2019 ▸) found an integral footprint in Africa over all use-cases for an AdLS to be more than 50% of Africa, where contributions are at least papers or theses. The AfLS Conference Series which had its sixth event on 13–17 November 2023 (AfLS6-2023, 2023 ▸) serves as a user meeting for the African synchrotron community.

## 3. A roadmap towards an AdLS for Africa

Africa’s timeline towards building its own AdLS is a story of ubuntu. In addition to its own set of challenges that need to be addressed, which give an African character to the roadmap towards the AfLS, there is the method by which Africa is communally undertaking this roadmap, together with all its partners. The spirit of ubuntu and an African identity have been reflected in the development of the vision as well as the early progress towards the AfLS in Africa.

Many of the indigenous African languages include a word for ubuntu.^1^ This is part of the African identity. There are others. Africa was the cradle of humankind, so we are all part of the first African diaspora. In the words of Kwame Nkrumah, one of the first Pan-Africanists, we should have a very inclusive view about who is an African. He maintained, ‘I am not African because I was born in Africa but because Africa was born in me’. So, although the AfLS is an African project, with African leadership and passion, the vision of the inclusivity of the African identity provides the space for the AfLS to be driven and led by several categories of visionaries: those resident in Africa, those from the various African diasporas, especially the current African science diaspora, and those who are friends of Africa worldwide. The context of this last category is that science can further diplomacy, democracy and world peace. That science is a global undertaking, all must contribute to the global scientific endeavour, and all must share in its fruits. The insistence on African passion also means that the AfLS venture can at most use major external funding in an initial transitional process, while the ultimate goal is for the major investment and operational funding to be sourced from within Africa itself.

A large-scale research infrastructure such as an AdLS should eventually be a largely Pan-African undertaking, even if crystallized initially via a core nucleus, then to become a large-scale community-driven project, that now soon is taken up by African national policy makers. Africa is a very large continent with exceptional diversity and a very complex history. Clearly, science diplomacy, both in the sense of science for diplomacy and diplomacy to enable science, will be an important approach to boost the AfLS venture, and have impact beyond the pure science case. The AfLS history is presented in this context in order to ensure that the spirit of ubuntu and the prevalence of an African identity persist throughout the next phases of the Pan-African AfLS project.

**Figure 1:**
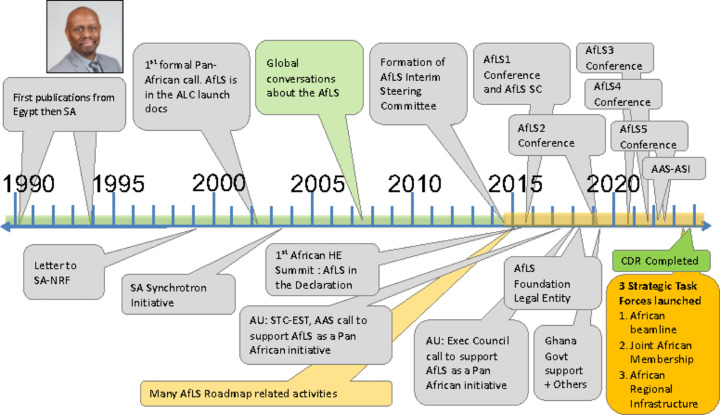
The timeline of progress towards the AfLS.

Fig. 1 ▸ displays the timeline of progress towards the AfLS. It is a summary of several references (Mtingwa & Winick, 2018 ▸; Connell *et al.*, 2018 ▸, 2019 ▸, 2023 ▸; Ngabonziza, 2019 ▸; Khan, 2022 ▸).

The first light-source-based papers by Africans date from the last decade of the previous century, as indicated in Fig. 1 ▸. Several groups were represented from Egypt and South Africa (Als-Nielsen *et al.*, 1994 ▸; Bhat *et al.*, 1990 ▸; Huisman *et al.*, 1997 ▸; Hearne *et al.*, 1996 ▸). At the same time, there began community-based and Africa-wide conversations, inclusive of all participants, both on and off the continent, involving individuals and organizations. These conversations concerned the advantages of light-source-based research, enhancing the participation of Africans, African capacity building, and also building the local African infrastructure which enabled successful proposals to international facilities. They constituted a growing groundswell of thinking that the time was ripe for Africa to consider its own AdLS. Some notable touch points are mentioned. The experience of Professor Hearne at the ESRF in 1996 (Hearne *et al.*, 1996 ▸) inspired him to write to the South African research funding agency, the National Research Foundation (NRF), advising a long-term goal for South Africa to construct a light source via a consortium of international partners, especially involving neighbouring countries in Southern Africa.

In 2000, the Edward Bouchet-Abdus Salam Institute (EBASI) issued a call to enhance local laser infrastructure towards a Pan-Africa light source. EBASI is an organization based at the Abdus Salam International Centre for Theoretical Physics (ICTP) in Trieste, Italy, that promotes African/African-American collaborations. This became the first formal call for an AfLS when it was included as a long-term goal in the founding documents of the *African Laser Centre – The Strategy and Business Plan* two years later (Mtingwa, 2013 ▸). Here Professor Sekazi Mtingwa played a leading role. Shortly after there were several articles on the need for an African synchrotron (Kebede *et al.*, 2003 ▸; Khan, 2003*a* ▸,*b* ▸,*c* ▸), which also proposed the African Synchrotron Research Programme. The AfLS had its origins in these and many other conversations, as they gained momentum towards the idea of a light source for Africa.

There also continued national efforts in different countries to highlight the role of the light source. In South Africa, Dr Tony Joel of Necsa, Gabriel Nothnagel and others wrote on this matter, and the South African Synchrotron Initiative (SASI) (Joel & Nothnagel, 2003 ▸; Joel, 2004 ▸) was formed. Also important in South Africa at this time, in 2004, are the documented deliberations of an expert international panel convened by the National Research Foundation (NRF) for the Science Ministry, tasked with Shaping the Future of Physics in South Africa (Hellberg *et al.*, 2004 ▸). Their report recommended a new flagship project with a synchrotron identified as an example for consideration. This led to government support of the South African Community to organize itself, resulting in several local synchrotron-based research-related conferences and workshops, a mobility grant facilitating access to international synchrotrons, and a Scientific Associate Membership by South Africa of the ESRF (Colvin, 2013 ▸).

The global conversations, writings and calls for an AdLS in Africa grew. The developments within each African country of smaller-scale local laboratory-based research exploiting similar, though not as optimal, techniques to those at large-scale AdLSs grew, as did the use of external national and international AdLS facilities. Of particular mention are the initiatives of the International Union of Crystallography (IUCr), the IUCr–UNESCO OpenLab project in a range of African countries (Zema & Lecomte, 2015 ▸; IUCr–UNESCO, 2023 ▸) and the X-TechLab project at Sèmè City in Benin (X-TechLab, 2023 ▸), which is an outgrowth of the Lightsources for Africa, the Americas, Asia, Middle East and Pacific (LAAAMP) programme (LAAAMP, 2023 ▸) in collaboration with the government of Benin. In the former OpenLab programme, multidisciplinary research programmes based on X-ray facilities, which could be newly acquired, already operational or used remotely, are developed at an existing centre of competency. In the latter programme, X-TechLab is a new development that promotes regional research excellence and capacity building in multidisciplinary research based around a new well equipped X-ray laboratory. These new facilities become a hub for regional excellence. In these examples, the local and regional facilities were well understood to also be part of a vision to grow the African user base at international light sources and ultimately to enhance the vision of an AfLS.

**Figure 2:**
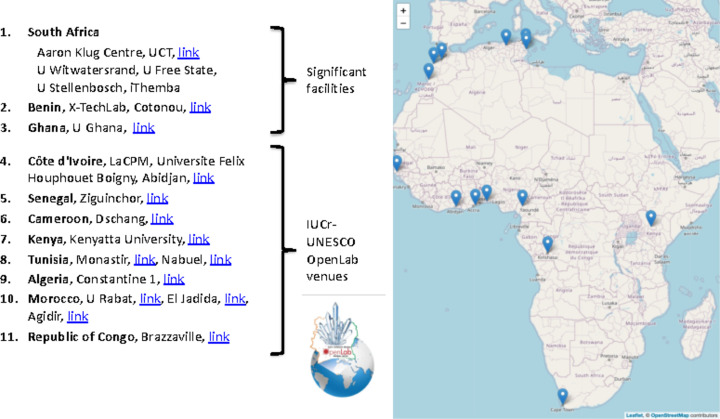
A survey of sites in Africa with X-ray-based research capacity in crystallography (The African Light Source, 2015 ▸).

Fig. 2 ▸ shows a survey of sites in Africa with X-ray-based research capacity in crystallography (The African Light Source, 2015 ▸). The same reference also shows the X-ray-based research capacity in the bio-sciences. This overview is not complete but serves to indicate the extent of the momentum building within Africa towards a joint Pan-African AdLS.

By 2015, global conversations about an AfLS had grown sufficiently, together with the growth in local and regional facilities, that there evolved a sufficient African footprint, such that it was decided the community-driven AfLS project should organize itself. This would be an ubuntu-driven bootstrap process. An Interim Steering Committee was formed to arrange the first African Light Source Conference, to convene in November 2015. There was a global effort to gather contact details of all possible participants and then to inform that community about the conference. The conference would showcase African light-source-based research, audit that, and also be an opportunity for the wider community, including international partners, to meet formally. The meeting convened at the ESRF as a result of an invitation by Director-General Francesco Sette, reflecting the assistance given to Africa by the international community for its first conference. Participants agreed that subsequent conferences would convene in Africa. The conference went beyond an academic programme and included a strategic and political component. It deliberated on procedures and structures for the AfLS project and then established these. For those who could not attend, early electronic contributions to the discussion and nominations were invited. The process allowed for maximum inclusivity, transparency and democracy. Accordingly, the General Assembly of this Conference established the fully mandated African Light Source (AfLS) Steering Committee. Finally, during the conference, the Interim Steering Committee dissolved itself, its work done, and was replaced by the new duly elected, fully mandated AfLS structures, which was several committees operating under the AfLS Executive Committee. The conference participants also developed the Grenoble Resolutions and the AfLS roadmap (AfLS Roadmap, 2023 ▸).

Following this, the AfLS Executive and its committees began to drive the process according to the roadmap. In 2018, the AfLS became a formal legal entity, the African Light Source Foundation. So far, there have been five subsequent AfLS conferences and workshops broadening the footprint of involvement and explicit support.

## 4. The scientific community and partner organizations

The AfLS is not the only group that promotes the vision of an AdLS for Africa. The large cohort of those who share, at least in some sense, the vision of the AfLS includes African researchers, Pan-African and African national voluntary professional research societies by discipline, some African academies, some African governments, the African Union, and many other internal and external stakeholders. The AfLS has developed partnerships with many of these. This relates to communication about activities, joint participation in conferences and joint strategic thinking and planning. The AfLS has a Memorandum of Understanding (MoU) with SESAME (SESAME, 2023 ▸), which is an international synchrotron light source in Allan, Jordan, with many member countries from the wider region. Another MoU is with LAAAMP (LAAAMP, 2023 ▸), which is a joint programme of the International Union of Pure and Applied Physics, International Union of Crystallography and the Abdus Salam International Center for Theoretical Physics. Many other organizations have sent letters of support (The African Light Source, 2015 ▸). The AfLS has collaborated with a number of Pan-African and African national science professional associations and science academies, including the African Physical Society (AfPS, 2023 ▸; Said *et al.*, 2020 ▸), Federation of African Societies of Chemistry (Abegaz, 2016 ▸), African Materials Research Society (Kebede & Msezane, 2001 ▸; Pelhan, 2015 ▸), Federation of African Societies of Biochemistry and Molecular Biology (Titanji, 2005 ▸), BioStruct-Africa (Ouologuem *et al.*, 2022 ▸; Nji *et al.*, 2019 ▸), Federation of African Immunological Societies (Sibanda & Barbouche, 2019 ▸; Osier *et al.*, 2020 ▸), Federation of African Medical Physics Organizations (Ige *et al.*, 2020 ▸), the very recently launched African Crystallographic Association (Roodt, 2017 ▸; Lecomte, 2017 ▸; Nyanganyura & Glover, 2017 ▸), East African Association for Palaeoanthropology and Palaeontology (Alemseged *et al.*, 2019 ▸) and Network of African Science Academies (Nakkazi, 2022 ▸; Crewe, 2016 ▸).

## 5. Science policy and diplomacy

**Figure 3:**
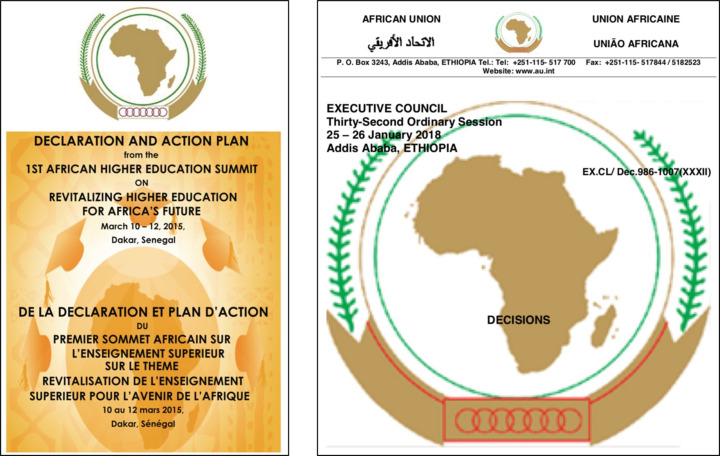
(Left) The Declaration and Action Plan of the 1st African Higher Education Summit on Revitalizing Higher Education for Africa’s Future (AUC, 2023 ▸) and (right) the 2018 Executive Council of the AU recorded a Decision on the Reports of the Specialized Technical Committees (STCs) item C for the STC on Education, Science and Technology and then Article 21, which ‘calls upon Member States to support the Pan-African Synchrotron Initiative’ (AU-EC, 2019 ▸).

One important Pan-African early deliberation on an AdLS for Africa was the occasion of the 1st African Higher Education Summit on Revitalizing Higher Education for Africa’s Future, which was held in Dakar, Senegal, during 10–12 March 2015. The summit was organized by several key Pan-African organizations, including the African Union Commission (AUC), TrustAfrica, Council for the Development of Social Science Research in Africa (CODESRIA), United Nation’s Africa Institute for Development and Economic Planning (IDEP), Association for the Development of Education in Africa (ADEA), Association of African Universities (AAU) and African Development Bank (AfDB). An outcome of the latter meeting was a Declaration and Action Plan (Fig. 3 ▸) to bring about a shared strategic framework for inclusive growth and sustainable development, and a global strategy to optimize the use of Africa’s resources for the benefit of all Africans. Article 5.3.3 on page 22 recommends establishing a synchrotron as a centralized African scientific facility.

**Figure 4:**
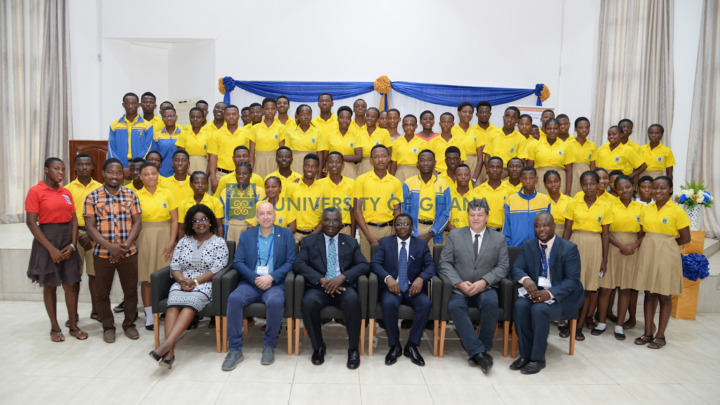
Meeting between AfLS, Pan African Conference on Crystallography (PCCr) and the Ghanaian Science Ministry during the joint AfLS–PCCr Conference in January 2019.

Following a 2018 presentation by the African Academy of Sciences (AAS), with input from the AfLS, to the African Union’s (AU) Specialized Technical Committee (STC) on Education, Science and Technology, the AU Executive Council issued a report calling upon member states to support the Pan-African Synchrotron Initiative (AU-EC, 2019 ▸). The AAS formed the Africa Synchrotron Initiative (AAS-ASI) in 2019, which began meeting as a one to two year think tank in 2022 (Khalil *et al.*, 2022 ▸; AAS, 2022 ▸). In January 2019, the AfLS Conference was held in Accra, Ghana. There were positive interactions with the Ghanaian Science Ministry (see Fig. 4 ▸) and a commitment from the President of Ghana, H. E. Nana Akufo-Addo, to be a champion of the African light source project, not only in his own government but also in the Economic Community of West African States and the AU (Ghana Business News, 2019 ▸).

## 6. The African community strategic thinking assessment

A recent assessment survey launched by the African Strategy for Fundamental and Applied Physics (ASFAP) Light Sources Working Group (ASFAP, 2021 ▸; Khalil, 2021 ▸; Fassi, 2021 ▸) was released in February 2023 to better realize the vision and the requirements of such a community towards establishing an African light source (Kamel, 2021 ▸). The survey gathered the thoughtful input of 225 professional participants from a very broad outreach to the African scientific community, among others, on the case of founding an African light source. We mention here only a brief summary of the survey results. 77% of the participants were resident citizens in African countries, while 26% were African diasporeans. Participants were from 19 African countries (Nigeria, Morocco, Kenya, Cameron, Senegal, South Africa, Ethiopia, Tunisia, Uganda, Algeria, Ghana, Sudan, Egypt, Ivory Coast, Zambia, Mozambique, Togo, Congo and Sierra Leone). Basic and applied science, life sciences, materials sciences, cultural heritage and archaeology, accelerator physics and technology, optical instrumentation, beamline development, as well as experimental instrumentation and data analysis were the scientific fields that were most highlighted. 70% of the survey’s participants demonstrated previous experience at light source facilities, whereas 76% expressed current and/or future synchrotron-related interests. This confirmed the inevitability of establishing such a facility in Africa. Geographical distribution, collaborations with other research institutions, access to remote databases and software, as well as advanced instrumentation were assigned as higher priorities for research issues requiring attention. 81% of participants marked the vital need for advanced training concerning the general use of similar and accessible infrastructures, together with financial, technical and scientific support. 88% of the participants expressed a willingness to initiate interactions on different axes of collaboration and assistance with other African groups. To conclude, there was a strong and evident intention to establish an African light source to deal with the major and extended challenges that Africa faces, for example, in the public health sectors.

## 7. Regional and Pan-African components of the AfLS roadmap

The AfLS is making excellent progress in the first part of its roadmap, which involves human capacity building activities and activities growing the user base of African scientists at AdLSs. Transitioning into the more mature part of the AfLS roadmap, introducing top-down elements, begins with the imminent release of the Conceptual Design Report. This identifies the scientific and socio-economic motivation, details the technological choices, specifies the scientific research goals, and initiates discussion on many other aspects, such as finance and governance. It is one of the important parts of the roadmap. In parallel with this are activities that require the increasing appreciation of the vital importance of an AdLS in Africa by African policymakers and higher-level political decision-making structures of African nations and Pan-African organizations. The more recent conferences of the AfLS have included African science ministers’ forums. The breadth and depth of the higher-level conversations have been increasing. This has led to the AfLS launching three new Strategic Task Forces, as detailed in the following.

### 7.1. African beamline at an international AdLS

This would be an African designed and operated beamline that could address selected African research imperatives. Additionally, it would lead to the training of engineers and technologists and would result in technology transfer.

### 7.2. Collective African membership of an international AdLS

Several African countries would jointly acquire formal membership of an international AdLS. They would leverage a threshold of participation that would allow African governmental involvement in the council of the facility, so that there is increased African access by researchers, technologists and industries.

### 7.3. African regional infrastructures

These are research infrastructures that are both highly competitive in their own right and important training and feeder infrastructure to an AdLS. Some current examples are the IUCr–UNESCO OpenLabs started in several African countries, X-TechLab in Benin and the Electron Microscope Unit in Cape Town, South Africa.

## 8. Conclusion

Synchrotron light sources represent one of the brightest examples of interdisciplinary as well as multidisciplinary research infrastructure. They deliver robust opportunities and practical solutions through cost-sharing, community networking and collaboration. Common environmental challenges such as air, soil and water pollution, then also food security and agriculture, also investment in advanced materials for solving energy complications, further, studies of human, animal, and plant health and diseases, also including cultural heritage conservation, are some of many complex issues that are demanding both in terms of fundamental science and connecting cross-disciplinary collaborations. In addition to the huge scientific value, light sources have also revealed their effectiveness in increasing employment rates and in reducing the gender gap; they represent a transparent and democratic environment that is based only on scientific merit and skills. Furthermore, they constitute an exercise in science diplomacy — based on scientific collaborations and relying on the neutral language of science. This, in turn, inspires and reassures the success of new and emerging partnerships on both the national and international levels to mutually tackle scientific and societal challenges, together with educational systems and economic development. Africa must not be considered an exception when it comes to the advanced scientific and technological arena in any of the above-mentioned examples. This is simply because Africa shares a concrete and tangible aspiration to see itself as an equivalent world leader, sharing the responsibilities together with the worldwide scientific endeavour.

The world is moving closer economically, intellectually and scientifically. International facilities similar to ESRF and SESAME can also be created in Africa. Given the cost and lead time in designing a new facility, we need to start preparing immediately. The voices for an AfLS date back at least two decades.

Research infrastructures are proven high-value pillars of modern knowledge-driven ecosystems. The number of such facilities in many domains is growing rapidly. Europe manages this via the European Strategy Forum for Research Infrastructures (ESFRI). ‘Landmark’ facilities, including the internationally supported ESRF, stand out as key elements of the European research agenda. Africa should, and must, develop similar approaches. Investment in an advanced light source, such as a synchrotron, should be a high-priority component of Africa’s drive to develop the continent as a world player in science and technology. Capacity building is underway, a roadmap is being pursued, and the vision for an African advanced light source by 2035 is entirely tenable.

## Footnotes

1 This describes a human community centred around values of inclusivity, fairness, tolerance, democracy, honesty, equity, consultation and empathy (Mugumbate & Chereni, 2020 ▸).
